# Death from a Bee Sting

**Published:** 1865-08

**Authors:** 


### Death from a Bee Sting.

MANSFIELD, OHIO, April 28, 1865.

Dr. Carpenter reports the following in the *Cincinnati Lancet and Observer:* "A very remarkable and fatal case of poisoning from the sting of the common honey bee, occurred near our city, on the 18th instant. The case is so unusual, I thought it best to make a note of the accident, and send the statement to you, for publication in the *Lancet.*

On the afternoon of the day above mentioned, I was summoned to the residence of Mr. John Krith, some two and a half miles north of our town to see his little son, four years old, who, I was informed, was suffering from spasms caused by the sting of a bee.

On arriving at the house, I was astonished to learn that the child had been dead thirty minutes, and was informed by the family that from the time the child was stung until his death, not over thirty minutes had elapsed. He had violent spasms from the first until he died.

The sting was immediately over the sagittal suture, one inch posterior to the articulation of the frontal with the parietal bone. There was but little redness, or swelling attending it. The mother had applied alkalies freely, but all to no avail.

Now the query naturally arises, in what way did the poison of that sting cause the death of the child so suddenly? Why should this terminate fatally so soon, when there are very many persons over the country every year, who are stung on various parts of the head, without experiencing any serious results?"

